# Nitrogen Balance at the Recommended Dietary Allowance for Protein in Minimally Active Male Vegans

**DOI:** 10.3390/nu15143159

**Published:** 2023-07-16

**Authors:** Eric Bartholomae, Carol S. Johnston

**Affiliations:** College of Health Solutions, Arizona State University, Phoenix, AZ 85004, USA; ebartho2@asu.edu

**Keywords:** nitrogen balance, vegan, protein requirement

## Abstract

Vegan diets have gained popularity in recent years for reasons including health benefits and concerns for animal welfare. Although these diets are considered to be nutritionally adequate, questions remain over whether the current protein recommendation (0.8 g/kg/d) is sufficient. Protein status is determined through a nitrogen balance analysis when the protein content of the diet is known. A negative balance indicates a catabolic state, and a positive nitrogen balance indicates an anabolic state. In healthy adults, nitrogen equilibrium is the expectation reflecting the net synthesis and breakdown of proteins. Currently, there are no known studies measuring nitrogen balance in strict vegan men fed the protein requirement. Eighteen minimally active vegan men received a 5-day eucaloric diet (protein content: 0.8 g/kg/d). On day five, 24 h urine was collected for nitrogen analysis. Both the mean absolute nitrogen balance (−1.38 ± 1.22 g/d) and the mean relative nitrogen balance (−18.60 ± 16.96 mg/kg/d) were significantly lower than zero (equilibrium) (*p* < 0.001). There were no correlations seen between nitrogen balance and age, years as vegan, or fat-free mass. Consuming 0.8 g/kg/d of protein is not adequate to produce nitrogen balance in men adhering to typical strict vegan diets for at least one year.

## 1. Introduction

In recent years, vegetarian diets have gained in popularity with 5% of United States (U.S.) adults aged 18–34 years old identifying as vegetarian, and half of those being vegan [1]. This amounts to 3.8 million vegetarians, nearly two million of whom are vegan. The popularity of vegetarian diets in the U.S. has been linked to ethical and environmental concerns, but the desire to have a healthier lifestyle was the most prominent reason for adopting vegetarianism [2]. Vegetarian diet adherence has been associated with reduced risk and mortality for several chronic conditions including cardiovascular disease and cancer [3,4]; however, vegetarian diet adherence is not consistently linked to reductions in all-cause mortality [5,6].

It is the position of the Academy of Nutrition and Dietetics that appropriately planned vegetarian diets are nutritionally adequate and appropriate for all stages of the life cycle [7]. Yet concern remains over the potential for inadequacies in several micronutrients, the omega-3 fatty acids, and protein [8,9,10]. Considering protein adequacy, the amino acid profile and the digestibility of dietary proteins differ between animal and plant sources. To support body protein synthesis, adequate amounts of indispensable amino acids must be ingested, e.g., those amino acids that cannot be synthesized in vivo. Many plant proteins have less optimal indispensable amino acid profiles in comparison with animal proteins [11]. Additionally, plant protein digestibility is reduced in comparison with animal proteins due to its structure and to the high levels of antinutritional factors present in many plants (e.g., protease inhibitors, insoluble fibers, and phytates) that interfere with the digestion and absorption of protein [12,13]. The digestible indispensable amino acid score [DIAAS] is the recommended manner for ranking the biological value of dietary proteins and is calculated using a protein’s amino acid profile and ileal digestibility [14,15]. Animal proteins such as dairy have higher DIAAS values (>100) than plant proteins such as pea, soy, or wheat (62, 84, and 45, respectively) [16].

Due to the lower biological value of plant proteins in comparison with animal proteins, greater intakes of plant proteins are necessary to meet protein synthesis needs. Tang et al. demonstrated that whey protein ingestion (10 g) was superior to an equal dose of soy protein for stimulating muscle protein synthesis at rest as well as following resistance exercise in young, healthy men (+18% and +31%, respectively) [17]. Gorissen et al. showed that casein protein ingestion (35 g) produced a higher postprandial myofibrillar protein synthetic response versus the same amount of wheat protein in healthy older men (+56%) and that greater amounts of wheat protein (60 g) were required to increase myofibrillar protein synthetic rates to those observed for 35 g of casein protein [18]. Generally, the literature supports an adequate protein status in U.S. vegetarians [19]; however, less information is available specifically for vegan-diet adherence. Moreover, much of the available data are from cross-sectional trials, and protein and energy intakes are not controlled; hence, it is difficult to assess whether the current protein recommendations are adequate for individuals following a vegan diet exclusively.

The recommended dietary allowance (RDA) for protein is 0.80 g/kg/d for all adults over 18 years of age, including vegetarians and vegans. The RDA is based on the results of numerous nitrogen balance studies, which are considered the gold standard criteria for determining protein requirements [20]. Protein is the only macronutrient containing nitrogen; hence, protein status is determined by comparing the amount of nitrogen ingested to the amount of nitrogen which is excreted. A negative balance indicates net protein catabolism, and a positive nitrogen balance indicates a net anabolic state. In healthy adults, nitrogen equilibrium is the expectation reflecting the net synthesis and breakdown of proteins. Rand and colleagues analyzed data from twenty-nine nitrogen balance subtrials (twenty-three mixed diets and six vegetable diets; *n* = 235) and concluded that the estimated RDA was 0.83 g/kg/d for all healthy adults regardless of age, gender, or diet group [19]. The authors stated that there were no significant differences in dietary protein source (e.g., animal vs. plant sources) with regards to protein needs [19]; however, the ‘plant-based diets’ included in this meta-analysis were defined as diets with “vegetable sources providing > 90% of total protein”, indicating that these diets could contain up to 10% animal protein. Furthermore, a close analysis of the data revealed an average nitrogen balance of −2.21 mg N·kg^−1^·d^−1^ and +7.39 mg N·kg^−1^·d^−1^ for the “vegetable diets” and mixed diets, respectively, and +5.41 mg N·kg^−1^·d^−1^ overall [19].

To date, a nitrogen balance trial in strict vegan participants has not been reported. The purpose of this study was to determine the nitrogen balance in minimally active, male vegans ingesting a controlled, eucaloric diet containing the protein RDA, 0.8 g/kg/d. It was hypothesized that participants would exhibit a negative nitrogen balance in response to this diet plan.

## 2. Materials and Methods

### 2.1. Participants and Study Design

Minimally active, male adults (20–45 years) who adhered exclusively to a vegan diet for at least one year were recruited from the Phoenix metropolitan area between October 2020 and October 2021 using fliers, word of mouth, email listservs, and local social media groups. Participants were healthy by self-report and minimally active (<150 min of moderate to vigorous exercise per week). Further exclusion criteria included prescription medications, muscle-building supplements, such as protein or creatine powders, food allergies, an unwillingness to consume the trial foods exclusively, and/or those participating in or training for competitive sports in the past year. The stage of the menstrual cycle can impact nitrogen balance; hence, women were not recruited for this trial [21,22]. This study was approved by the Institutional Review Board at Arizona State University (STUDY00012662), and all participants provided written informed consent.

Following an initial screening via an internet questionnaire, potential participants took part in a separate, follow-up phone screening to explain to them the procedures and requirements of the study and ask any follow-up screening questions that may need clarification by the investigator. Upon agreement, qualifying participants were then scheduled to visit the lab where written informed consent was obtained. At the visit, anthropometric data (height, body mass, and waist circumference) were collected. Fat-free mass was assessed via bioelectrical impedance analysis following a 12 h fasting period in which no food, beverages, or water were consumed [23]. Participants received all foods for a complete 5-day menu plan personalized to provide maintenance energy for light activity (Harris–Benedict equation × 1.3) and 0.8 g/kg protein. Diets consisted of frozen meals, meal replacement shakes, and dried fruits with protein from mixed, complementary plant-based sources of varying degrees of protein quality based on DIAAS values, held constant at 0.8 g/kg/d (See Table 1). Participants were also allowed to eat selected foods from a list of fruits and vegetables, and logged all foods eaten in a daily food log (Table 2). Participants were scheduled to begin the 5-day feeding period immediately following the baseline visit.

Participants were provided instructions and a container for the 24 h urine collection on day 5, the final day of feeding. During the 5-day feeding period, participants were asked to refrain from any moderate–vigorous physical activity and limit all other activities in general. Participants tracked activity daily using the validated Godin leisure time physical activity questionnaire, and a score ≥24 METs × hours/week was the cutoff for ‘active’ [24]. Participants were instructed to record any uneaten food portions or additional foods eaten. For the 24 h urine collection, the first morning void was discarded, and all urine was collected throughout the day and overnight, including the first morning sample on day 6. No urine preservative was necessary and participants were asked to refrigerate the sample. The 24 h urine sample was delivered to the lab on the morning of day 6.

### 2.2. Diet and Urine Analysis 

Diet records were reviewed with the participants on their return to clarify any ambiguities and were analyzed by a trained investigator using the Food Processor software (version 7.71; ESHA Research, Salem, OR, USA). Urine samples were thoroughly mixed, total volume determined, and aliquots frozen at −80 °C for later analysis via photometric assay to determine nitrogen content by Sonora Quest Laboratories. Nitrogen balance was determined using the known protein content of the diet on the fifth day of consumption and the nitrogen content of the urine (as UUN) using the equation

Nitrogen Balance (g/d) = (PRO intake (g/d)/6.25) − UUN (g/d) − 4

where the coefficient of 6.25 is derived from the knowledge that protein is 16% nitrogen (e.g., there are 6.25 g of nitrogen per g of protein) [25]. A constant of 4 is used to account for obligatory nitrogen losses: 2 g urinary non-urea nitrogen excretion (e.g., ammonia, uric acid, creatinine, and amino acids) and 2 g gastrointestinal, integumentary (dermal), and sweat losses [26].

### 2.3. Statistical Analysis

Data for this cross-sectional study are reported as the mean ± SD, and an a priori α of 0.05 used to determine significance. All outcome data were tested for normality and nonparametric statistics used when necessary. Statistical analyses were performed using SPSS version 27 (IBM, Armonk, NY, USA). To determine if calculated nitrogen balance values were different from zero (nitrogen equilibrium), a one-sample *t*-test was used. Simple regression analyses were used to understand whether nitrogen balance could be predicted based on diet duration, age, fat free mass, or physical activity (Spearman rank test). Sample size was determined using G*Power version 3.1 (Heinrich Heine Universität, Düsseldorf, Germany). Based on Rand et al. [19], given an expected change of 5 mg nitrogen/kg/d and an SD of 6.4 mg nitrogen/kg/d, this yields an effect size of 0.78 (e.g., 5/6.4 = 0.78). Using a predetermined α of 0.05, a sample size *N* = 16 yielded a power of 90%. Thus, allowing for an attrition rate of 20%, 20 participants was the enrollment goal.

## 3. Results

### 3.1. Participant Characteristics

**Figure 1:**
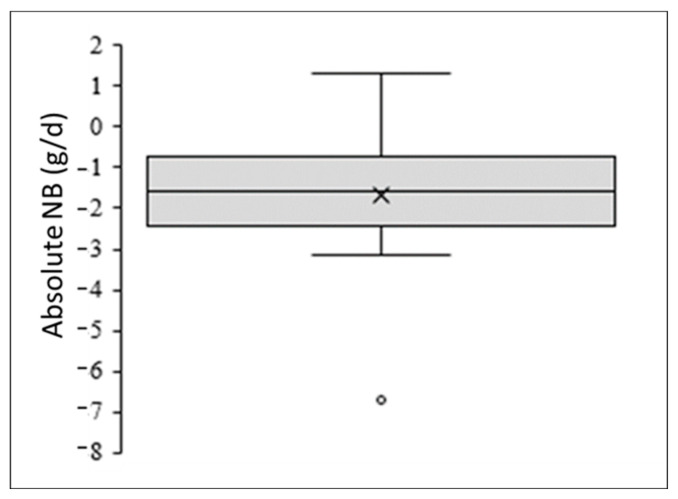
Nitrogen Balance (NB) (g/d) Box Plot Analysis displaying the median (×) and the outlier value (◦).

One hundred and twenty people responded to the online screening questionnaire, and thirty-five met the eligibility criteria for enrollment. Twelve of these qualifiers did not respond to emails, three declined to participate after a phone interview, and two withdrew from the study prior to any participation. Thus, 18 participants were enrolled and completed the study. Prior to analyses, age, years vegan, fat-free mass, physical activity, and nitrogen balance values were assessed for normality and potential outliers. A box plot analysis determined that only nitrogen balance had an outlier which was confirmed by Shapiro–Wilk normality testing (W(18) = 0.89, *p* < 0.047) (Figure 1). This participant was removed from all analyses, and data are presented for 17 participants.

Participants were young healthy male adults aged 31.6 ± 6.2 years (range: 25–43 years; body mass index: 24.2 ± 3.8 kg/m^2^) (Table 3). Four participants were overweight (BMI 25.0–29.9 kg/m^2^) and two were obese (BMI > 30.0 kg/m^2^). Adherence to the vegan diet averaged 7.1 ± 6.5 years (range: 1–23 years). Maintenance energy was calculated using the Harris–Benedict equation, accounting for light activity (2377 ± 362 kcal) and protein needs (0.8 g/kg/d) averaged 60.9 ± 10.5 g/d (Table 3).

### 3.2. Nitrogen Balance

**Figure 2:**
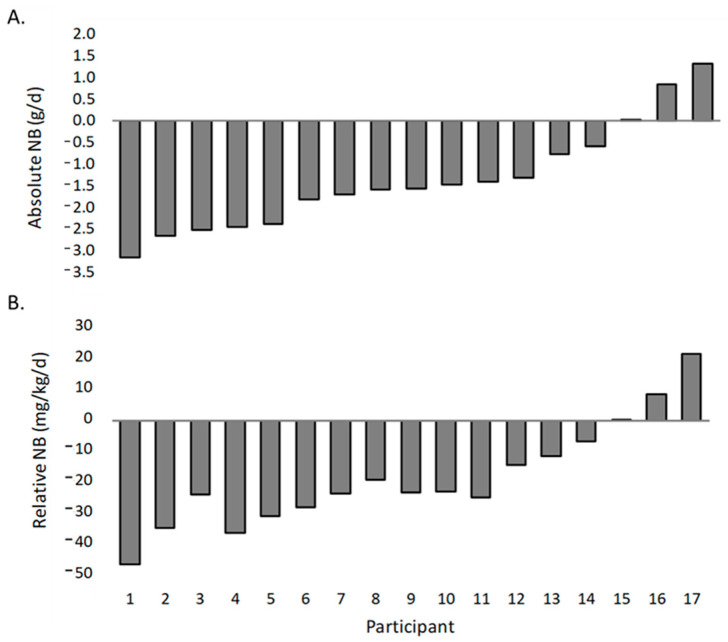
Individual participant nitrogen balance (NB) data: (**A**) Absolute NB (g/d) and (**B**) Relative NB (mg/kg/d).

A one-sample *t*-test was performed to determine whether nitrogen balance values following the 5-day dietary protocol were statistically different than nitrogen equilibrium (a nitrogen balance value of zero). Nitrogen balance was analyzed as absolute nitrogen balance in grams per day (g/d) and, relative to body weight, as relative nitrogen balance in milligrams per kilogram per day (mg/kg/d). The mean absolute nitrogen balance (−1.38 ± 1.22 g/d) was statistically lower than the nitrogen equilibrium score of zero [(95% CI, −2.00 to −0.75), *t*(16) = −4.643, *p* < 0.001]. The mean relative nitrogen balance score (−18.60 ± 16.96 mg/kg/d) was statistically lower than the nitrogen equilibrium score of zero [(95% CI, −27.32 to −9.88), *t*(16) = −4.522, *p* < 0.001]. Individual participant nitrogen balance values are displayed in Figure 2A,B).

There were no significant correlations between nitrogen balance and age, years vegan, fat-free mass, BMI, physical activity, or other descriptive variable (*p* ≥ 0.100; Spearman rank correlation). A weak correlation was noted for age (r = −0.409; *p* = 0.103). The ages for the two participants displaying a positive nitrogen balance were 25 and 26 years. Daily energy intakes during the trial ranged from 1925 to 3231 kcals (average, 2338 ± 380 kcals/d; calculated average energy needs pre-trial, 2377 kcals/d), and the daily protein intake averaged 61 ± 9 g (calculated average protein need pre-trial, 60.9 g/d). Nitrogen balance was not related to energy or protein intakes.

## 4. Discussion

The U.S. protein RDA for adults (0.8 g/kg/d) is defined as the amount to achieve a ‘zero nitrogen balance’ [27]. The data presented herein suggest that 0.8 g/kg/d was not adequate to maintain nitrogen balance in men who have adhered to a strict vegan diet for at least one year. The U.S. protein requirement was informed by a series of nitrogen-balance studies systematically reviewed by Rand et al. [19], which incorporated trials focused mainly on animal proteins (trials = 23, participants = 247). Of the few trials that fed predominately plant-based proteins (trials = 6, participants = 73), animal proteins made up to 10% of the dietary protein in these trials, and participants were omnivores who only omitted animal foods for the purpose of these trials [19]. The present results, when compared with the plant-based protein data presented by Rand et al., showed a markedly decreased relative nitrogen balance (−18.60 ± 16.96 mg/kg/d versus −2.2 ± 7.75 mg/kg/d, respectively). Putting these results into perspective, a mean nitrogen balance of −1.38 g/d signifies a daily loss of 8.63 g body protein, or a loss of 3.1 kg body protein over one year.

Previous research that used 4-day diet recalls to calculate the protein digestibility corrected amino acid scores for the diet plans of 22 vegetarian women suggested that the protein requirement for vegetarians appeared to be 25% higher than that for omnivores [10]. In the present study, about 12 g of additional protein (incorporating a 74% digestibility factor for vegan diets [28]) is needed to counter the nitrogen losses, equating to a 20% increase in the protein requirement (e.g., 0.96 g/kg/d).

Nitrogen balance trials in vegetarian populations are scarce; however, several early studies support a protein requirement of 1 g/kg/d for individuals adhering to vegetarian diets. Yanez et al. [29] conducted a long-term nitrogen balance trial in eight Chilean men fed a controlled, eucaloric vegetarian diet for three months with protein at 1 g/kg/d. Body weight remained stable during the trial, and nitrogen balance averaged +6.7 mg/kg/d. Only one of the eight participants recorded a negative relative nitrogen balance (−2.3 mg/kg/d). Register et al. [30] also reported an overall positive nitrogen balance (+0.07 g/d) in six young adults fed a vegan, eucaloric diet for nine days with protein held at 1 g/kg/d; however, three of six participants reported a negative nitrogen balance (average: −3.4 g/d).

Negative nitrogen balance indicates a decline in body protein mass. For a healthy inactive adult consuming a eucaloric diet at the RDA of 0.8 g/kg/d, a negative nitrogen balance would suggest inferior protein quality and a lack of essential amino acids rather than an inadequate quantity of protein [31]. A chronic negative nitrogen balance would adversely impact the synthesis of new proteins and eventually reduce skeletal muscle mass and the synthesis of enzymes, hormones, and immune factors, and impede tissue maintenance and repair [32,33]. Negative nitrogen balances are noted in clinical cases including individuals with protein energy malnutrition and older adults suffering from sarcopenia [34,35]. Although muscle loss has not been studied over the long term in vegetarians, age-related muscle loss begins at about age 40, and up to 50% of muscle mass may be lost by age 80 [35,36]. The loss of muscle mass in older adults has been linked to lower protein intakes [37], and experts recommend a higher protein intake for older adults (1.0 to 1.5 g/kg/d) to combat muscle loss [38,39].

Vegan diet adherence has been linked to lower muscle mass in young adults in cross-sectional trials. Vanacore et al. [40] showed a nearly 5 kg difference in muscle mass between omnivore (*n* = 10; 32.1 ± 0.81 kg) and long-term vegan (*n* = 10; 27.3 ± 1.2 kg) cohorts, with no difference between vegetarians (*n* = 10; 32.8 ± 1.4 kg) and omnivores. The study sample had a mean age of 29 ± 5 years and participants were age matched. Another study examined the relationship between protein intake from animal-based sources (1.05 g/kg/d) versus plant-based sources (0.98 g/kg/d) and muscle mass in healthy women [41]. They found that the vegetarian group (*n* = 19, with only one strict vegan; mean age = 48 ± 12 years; mean years on diet = 12) had significantly lower muscle mass compared with the omnivore control group (*n* = 21) (18.2 ± 3.9 kg vs. 22.6 ± 5.0 kg, respectively). There was also a significantly lower muscle mass index in vegetarians (6.7 ±1.2 kg/m^2^) compared with omnivores (8.3 ± 1.5 kg/m^2^). The data suggested that animal protein intake was an independent predictor of muscle mass index (adjusted r^2^ = 0.42) [41]. Caso et al. demonstrated that albumin synthesis was lower in participants adhering to a vegetarian versus omnivore diet plan when protein intakes were held at 1 g/kg/d [42]. However, when supplementing 18 g soy protein to the vegetarian diet plan (raising protein intake to 1.25 g/kg/d), the albumin synthesis rate balanced between diet groups [42]. Monteyne et al. [43] randomized participants to two eucaloric-controlled diet groups (omnivore versus strict vegan) for 3 days (protein = 1.8 g/kg/d) and reported similar myofibrillar protein synthesis rates between diet groups for both rested and exercised muscle. Based on these results, as recommended for older adults to combat sarcopenia, vegetarian populations should be advised to consume protein at levels above the RDA (e.g., 1.2 to 1.8 g/kg/d).

The analyses of possible predictors of nitrogen balance in the present study (e.g., age, years vegan, FFM, or physical activity) yielded no significant relationships suggesting that minimum protein requirements are not influenced by these factors in healthy adult men, assuming overall energy intake is adequate and physical activity is minimal. Nitrogen balance is sensitive to variations in energy intake, and energy intakes were tightly controlled in the present trial. Basal metabolic rates were calculated for each participant and personalized menu plans were devised to provide energy to support light activity (basal metabolic rate × 1.3). All foods were provided during the 5-day feeding period, the minimum adaptation period recommended for protein equilibration [19]. Participants were mostly inactive prior to the study and were asked to remain sedentary during the study protocol to standardize physical activity.

Further strengths of this study include personalized menu planning to incorporate mixed, complementary proteins from various plant-based sources to reflect participants’ normal diets, thus enhancing ecological validity (e.g., dietary protein was not limited to a single protein source). Importantly, this study was limited to long-term (>1 year), strict vegans, for which data in the literature was previously lacking. The current U.S. protein RDA was determined using nitrogen balance studies in omnivores and a few vegetarians—all of whom consume animal products, at least in part. The results of this study will serve as a starting point to better inform protein recommendations for those following a vegan lifestyle.

This study had several limitations. First, this was a short-term study, with an adaptation feeding period of five days. This is at the short end of what is considered adequate for nitrogen balance determination with standard protocol being between four days to several weeks adaptation to the experimental diet before the measurement of nitrogen balance [19]. Next, this was not an inpatient study, thus participants were free to live their daily lives during the study protocol. Because of this, dietary menu adherence was based on participants’ trustworthiness in tracking their intake. Although all food was provided to participants at no cost, it is possible they may not have consumed all meals, added prohibited foods, or measured certain foods incorrectly, thus providing inaccurate information on total energy and protein intake. Like dietary intake, physical activity was based on participants’ honesty of tracking outside of the laboratory as well. While participants were asked to keep all physical activity to a minimum, this definition may vary between individuals and can affect energy needs, thus nitrogen balance. Future studies of a similar design should better assess physical activity using body worn accelerometers to gather objective data. This study utilized a single protein intake for all individuals in order to test the adequacy of the U.S. protein RDA for a vegan population. While our data shows that the RDA was inadequate in achieving nitrogen balance, it is unknown at what intake this equilibrium could be achieved. Future studies could employ a crossover design, with participants consuming other protein amounts such as 1.0 g/kg/d, 1.2 g/kg/d, and so on in a randomized order to better determine protein adequacy. Lastly, this study used only a small sample of underactive males, thus these results are not generalizable to females or those engaging in physical activity or exercise. Further work is needed in these areas.

## 5. Conclusions

The results of this study suggest that the current U.S. RDA for protein, 0.8 g/kg/d, is not adequate to produce nitrogen equilibrium in underactive, vegan males consuming typical menu plans. This is important, given the increasing numbers of people who follow a vegan lifestyle, and the fact that high quality and quantity protein foods are less common in a vegan diet compared with an omnivore diet. Due to the inferior protein quality of most plant-based foods compared with animal-based foods, it is likely that the protein RDA should be amended with special recommendations for vegans, or at the very least highlight the importance of a diet with a higher protein intake to better guide and assure nutrient adequacy in vegans.

## Figures and Tables

**Table 1:** Example Meal Plans for Vegan Diets *.

Vegan Example Menu			
		**Protein (g)**	**Energy (kcal)**
Breakfast	Orgain Protein Shake	20	150
	Cinnamon Raisin Bagel	9	280
	Peanut Butter (2 T)	8	180
	Apple	0	65
Lunch	Amy’s Indian Vegetable Korma	9	330
	Dried Pineapple (2 servings)	0	280
Dinner	Sweet Earth General Tso’s Tofu	10	330
	Trader Joes Dried Mango (1/2 package)	0	280
	Peanut Butter (2 T)	8	180
	Fruit Snacks (2 packages)	0	160
Total		64	2235

* Example meal plan is for 80 kg male using the Harris–Benedict equation and a physical activity factor of 1.3 (seated work, no purposeful exercise). Meal plans provided 0.8 g protein/kg/d. Frozen entrees from Amy’s Kitchen and Sweet Earth.

**Table 2:** List of Permitted Foods (≤3 servings/d) to Supplement Diet Plan.

2 large celery stalks
2 cups shredded romaine lettuce
½ cucumber
1 medium tomato
½ cup sugar snap peas
1 carrot
1 cup jicama sticks
1 peach
½ grapefruit
1 cup winter mix vegetables
1 cup Tuscan-style vegetables
1 cup mixed broccoli, cauliflower, and carrots
¾ cup whole green beans

**Table 3:** Participant Characteristics ^1^.

	*N* = 17
Age (years)	31.6 ± 6.2
Years vegan (years)	7.1 ± 6.5
Height (cm)	176.6 ± 8.1
Weight (kg)	75.5 ± 13.7
Waist circumference (cm)	85.9 ± 10.5
Hip circumference (cm)	101.3 ± 8.3
Fat-free mass (kg)	60.0 ± 7.5
Fat mass (kg)	15.1 ± 8.2
Body fat (%)	19.2 ± 7.2
Body mass index (kg/m^2^)	24.2 ± 3.8
Physical activity (METs × hours/week)	18.1 ± 28.2
Maintenance energy (kcal/d)	2377 ± 362
Protein requirement (g/d)	60.9 ± 10.5
Nitrogen balance (g/d)	−1.38 ± 1.22 *
Nitrogen balance (mg/kg/d)	−18.60 ± 16.96 *

^1^ Data are mean ± SD. Maintenance energy for light activity was calculated using the Harris–Benedict equation × 1.3. Protein requirement, 0.8 g/kg/d. Asterisk indicates significant difference from nitrogen equilibrium (*p* < 0.05; one sample *t*-test).

## Data Availability

Data will be shared upon request.
